# Pulmonary Vein Isolation in Elderly Patients With Atrial Fibrillation and Symptomatic Sick Sinus Syndrome: A Case Series

**DOI:** 10.1111/pace.70170

**Published:** 2026-02-20

**Authors:** Cerine Benachi, Patrick Nagel, Johannes Lucas, Ulf Landmesser, Gerhard Hindricks, Verena Tscholl, Sebastian Biewener, Martin Huemer, Philipp Attanasio, Tobias Schreiber

**Affiliations:** ^1^ Klinik Für Kardiologie, Angiologie und Intensivmedizin Deutsches Herzzentrum der Charité Berlin Deutschland; ^2^ German Centre for Cardiovascular Research (DZHK) Berlin Germany; ^3^ Berlin Institute of Health (BIH) Berlin Germany; ^4^ Vivantes Auguste‐Viktoria‐Klinikum Berlin Germany

**Keywords:** atrial fibrillation, catheter ablation, internal loop recorder, pacemaker implantation, sinus node disease

## Abstract

In patients with atrial fibrillation (AF), sinus node disease (SND) represents a potentially reversible concomitant condition. Initial treatment with catheter ablation (CA) aimed at restoration of sinus rhythm may provide an alternative to pacemaker implantation. In this case series, 15 elderly patients (>65 years) with AF and SND underwent CA, with follow‐up monitoring provided by implantable loop recorders (ILR). Despite CA of AF, pacemaker implantation was necessary in 40% of patients during a mean follow‐up period of 37 months.

## 1 Introduction

Upon conversion into sinus rhythm, atrial fibrillation (AF) can lead to symptomatic pre‐automatic pauses, a subtype of sinus node disease (SND) [1]. Treatment of AF with catheter ablation (CA) may prevent SND progression through improvement of sinus node function and elimination of preautomatic pauses [2]. Furthermore, withdrawal of rate‐controlling agents after successful CA can improve sinus bradycardia and sinus pauses.

As an alternative strategy to pacemaker implantation, the current 2024 ESC guidelines recommend CA in selected cases (Class IIA, Level C) [3]. Data to support this strategy is derived from four non‐randomized trials [2, 4, 5, 6]. In these trials, the patient population had a mean age of less than 65 years, and follow‐up was conducted using Holter ECG. However, as age is a relevant risk factor for both arrhythmia recurrence and SND, it is unclear whether the results of those trials apply to elderly patients [7, 8].

We present a case series where CA was chosen as initial treatment approach for elderly patients with AF and SND, with follow up available via implantable loop recorder (ILR).

## 2 Case Series

Patients who met the following inclusion criteria were retrospectively identified:

- CA of paroxysmal or persistent AF
- first‐time diagnosis of symptomatic SND, either as preautomatic pauses upon AF termination or concomitant sinusarrest/sinoatrial block, all defined with pauses ≥ 3 s and symptoms (presyncope, syncope)
- no further indication for pacemaker implantation (preserved AV‐conduction)
- implantation of a loop recorder and
- age ≥ 65 years.

After CA and ILR implantation, patients were included in the remote monitoring system to enable continuous follow‐up. Endpoints were post‐ablation freedom from symptomatic bradycardia and overall arrhythmia freedom. The case series was permitted by the local ethics committee of Charité‐Universitätsmedizin Berlin (EA1/131/24).

In total, 15 patients were included with a mean age of 77.3 ± 4.9 years and mean follow‐up duration of 37.7 months (±83.9). Ten patients (67%) had preautomatic pauses, five patients (33%) had symptomatic pauses in sinus rhythm (sinoatrial block or sinusarrest). Mean duration of pauses was 5.7 s (SD 3.0). Baseline characteristics are shown in Table 1. CA was successfully performed in all patients; one groin hematoma occurred, which resolved spontaneously. Furthermore, one patient had early AF recurrence (two days after CA) and underwent electrical cardioversion.

**TABLE 1:** Patient characteristics.

BMI, mean SD (kg/m^2^)	Female, *n* (%)	Coronary heart disease, *n* (%)	COPD, *n* (%)	DMT II, *n* (%)	Arterial hypertension, *n* (%)
26.6 ± 5.6	10 (67)	8 (53)	3 (20)	3 (20)	12 (80)
**Moderate to severe mitral regurgitation, *n* (%)**	**Moderate to severe aortic regurgitation, *n* (%)**	**Paroxysmal atrial fibrillation, *n* (%)**	**CHADS‐Va (median; IQR)**	**Duration of atrial fibrillation, (months; median, IQR)**	**GFR (ml/min; mean+SD)**
5 (33)	3 (20)	10 (67)	3 (2)	5.5 (58.5)	57.7 ± 20.1
**NT pro BNP (mean+SD)**	**QRS duration before CA (median, IQR)**	**PQ‐intervall before CA, (ms; mean+SD)**	**LVEF (%; median; IQR)**	**TAPSE (mm, mean±SD)**	**Left atrial diameter (mm, median; IQR)**
1601 ± 1256	85.0 (39.5)	166.2 ± 21.2	55.0 (6.5)	20.7 ± 4.4	42.0 (6.0)
**First‐time CA, *n* (%)**	**PFA/Cryoablation, *n* (%)**	**Radiofrequency ablation, *n* (%)**	**LVA > 35%, *n* (%)**	**Heart‐rate controlling agents before CA, *n* (%)**	**Heart‐rate controlling agents after CA, *n* (%)**
9 (60)	5 (33) / 1 (7)	9 (60)	3 (20)	11 (73)	6 (40)

Abbreviations: COPD, Chronic Obstructive Pulmonary disease; DMT II, Diabetes Mellitus Type II; TAPSE, Tricuspid Annular Plane Systolic Excursion; CA, Catheter Ablation; PFA, Pulsed Field Ablation; LVA, Low Voltage Area.

**FIGURE 1:**
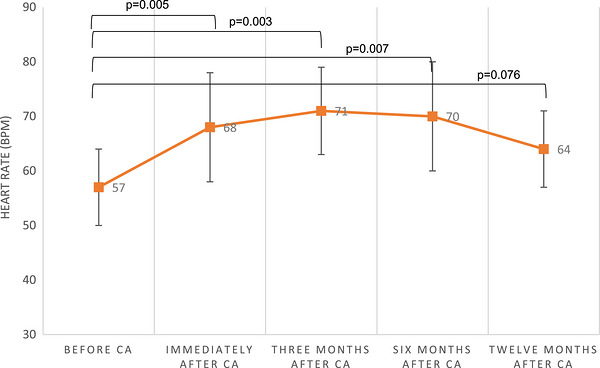
Mean heart rate with SD before, immediately (one to three days) after ablation, and during follow‐up. CA, Catheter ablation. [Colour figure can be viewed at wileyonlinelibrary.com]

Mean heart rate increased significantly shortly after as well as three and six months at follow‐up after CA compared to before CA (see Figure 1). Rate‐controlling agents were present before CA in 11 cases (73%) and continued with the same dosage in two cases (13%) and with lower dosage in four cases (27%). Antiarrhythmic therapy (amiodarone) was started in one case with early (one day after CA) AF recurrence.

Six patients (40%) required permanent pacemaker implantation. Three patients had continuous, symptomatic sick sinus syndrome; in three other patients, sinus rhythm could not be established after the first ablation procedure, and AF with symptomatic preautomatic pauses reoccurred. Pacemaker implantation was performed after a mean of 143 days (SD 168) after the initial ablation, considering the clinical situation and the patients’ preference. At the time of the first interrogation performed six to eight weeks after implantation, mean atrial pacing percentage was 39% (± 40.0); ventricular pacing percentage was low with a mean percentage of 0.1% (± 0.2). Rate‐controlling agents were restarted in five cases, antiarrhythmic drugs were used in two cases. Mean AF burden was 20.7% (+18.0).

**FIGURE 2:**
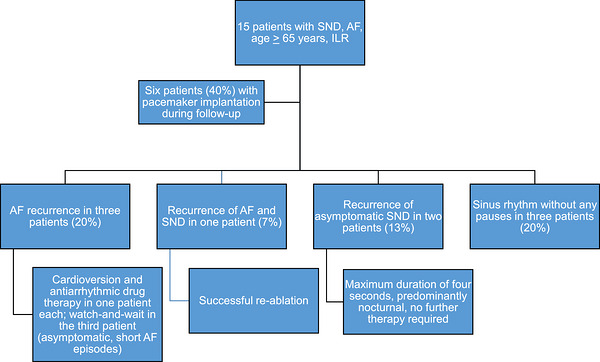
Follow‐up of patients without need for pacemaker implantation. AF, Atrial fibrillation; ILR, Implantable loop recorder; SND, Sinus node disease. [Colour figure can be viewed at wileyonlinelibrary.com]

Overall, only three patients (20%) remained in sinus rhythm without any pauses or arrhythmia recurrence (see Figure 2).

## 3 Discussion

In this case series, we evaluated for the first time the recurrence of AF and the progression of SND in elderly patients undergoing CA for AF using continuous ILR monitoring. The 2024 ESC guidelines for AF cite four studies [2, 4, 5, 6], in which fewer patients (4.7%–11%) underwent pacemaker implantation. Mean age was ≤ 65 years, comorbidities such as coronary heart disease (5.4%–25%), arterial hypertension (38%–56%), left atrial dilatation (diameter 36.3 to 38.1 mm) were lower compared to our cohort. These circumstances may at least be partially accountable for the higher AF and SND recurrence in our patient cohort. Other studies report pacemaker implantation rates from 7.4% [9], 9% [10], 13.8% [11] and 14.7% [12] with a maximum average patient age of 70 years [11].

### 3.1 Choice of Ablation Modality in Patients With AF and Concomitant SND

The choice of ablation modality has important implications in patients with AF and concomitant SND. Evidence suggests that radiofrequency ablation can improve sinus node recovery time as well as mean and maximal heart rate [2]. However, cryoballoon ablation has a more pronounced ability to modify autonomic cardiac input, including the disruption of vagal ganglionated plexi which project parasympathetic nerve fibers towards the sinus node [13, 14]. Consequently, studies have demonstrated increased heart rate after cryoballoon ablation [15, 16]. The extent of heart rate increase seems to be highest during ablation of the right superior pulmonary vein and is more pronounced in patients with pre‐interventional bradycardia [13]. However, long‐term follow‐up in the CIRCA‐DOSE (Cryoballoon vs Irrigated Radiofrequency CA: Double Short vs Standard Exposure Duration) trial suggests no lasting influence on heart rate and autonomic parameters regarding ablation modality [16]. In our case series, we observed similar results.

In patients with AF and concomitant SND, the differential autonomic impact across ablation modalities should be considered and thermal ablation might offer a relative advantage over pulsed field ablation, as demonstrated in a recent metaanalysis [15]. However, no direct head‐to‐head data comparing ablation modalities with respect to pacemaker implantation rates have been published.

### 3.2 Predictors of Pacemaker Implantation After CA of AF

Several clinical markers have been associated with a significant risk of pacemaker implantation after CA in patients with concomitant SND. These are derived from retrospective studies and include age, scarred left atria [17], left atrial dilatation [11], length of pauses [18] as well as female gender, structural heart disease and impaired LVEF [12]. Early recurrence of AF was also established as risk marker [12] for future need of pacemaker implantation. If CA is the chosen treatment modality to prevent pacing, it should be performed early after diagnosis of AF/SND, to enable reverse remodeling of the sinus node [19]. Patients with relevant risk factors should be closely monitored, and when restoration of sinus rhythm by means of pulmonary vein isolation seems no longer feasible, pacemaker implantation plus antiarrhythmic therapy may be considered.

So far, there is no prospective, randomized data comparing the effectiveness of both strategies. However, in patients undergoing pacemaker implantation as primary strategy, a meta‐analysis including four retrospective studies found that tachycardia‐related hospitalization was higher while in patients with CA, sinus rhythm was more frequent [10].

## 4 Conclusion

In this case series with elderly patients presenting with symptomatic SND and AF, CA was able to prevent pacemaker implantation in 60%. Continuous follow‐up is recommended in patients at risk for progression of SND and arrhythmia recurrence.

## Author Contributions

Philipp Attanasio – data collection and interpretation, critical revision and approval of article. Cerine Benachi – data collection, data analysis, statistics, drafting article. Sebastian Biewener – concept, data collection, critical revision and approval of article. Gerhard Hindricks – critical revision and approval of article. Martin Huemer – critical revision and approval of article. Ulf Landmesser – critical revision and approval of article. Johannes Lucas – data collection, critical revision and approval of article. Patrick Nagel – data collection, critical revision and approval of article. Tobias Schreiber – concept, data collection, data analysis, statistics, drafting article, critical revision of article. Verena Tscholl – critical revision and approval of article.

## Conflicts of Interest

The authors declare no conflicts of interest.
